# Efficient gene transfer by pulse parameters for electrochemotherapy of cells *in vitro* and in muscle and melanoma tumors in mice

**DOI:** 10.2478/raon-2025-0027

**Published:** 2025-04-21

**Authors:** Masa Omerzel, Bostjan Markelc, Simona Kranjc Brezar, Gregor Sersa, Maja Cemazar

**Affiliations:** 1 Department of Experimental Oncology, Institute of Oncology Ljubljana, Ljubljana, Slovenia; 2 Faculty of Health Sciences, University of Ljubljana, Ljubljana, Slovenia; 3 Biotechnical Faculty, University of Ljubljana, Ljubljana, Slovenia; 4 Faculty of Health Sciences, University of Primorska, Izola, Slovenia; 5 University of Ljubljana, Slovenia

**Keywords:** gene electrotransfer, electrochemotherapy, cells, tumor, muscle

## Abstract

**Background:**

In recent years, various gene therapy strategies have been developed for cancer treatment. One of these strategies is electroporation-based delivery of therapeutic transgenes - gene electrotransfer (GET). Electrochemotherapy and GET have been combined in several contemporary preclinical and veterinary studies. In most cases, two different pulse protocols are used, each for a specific treatment. The aim of our current study was to test whether the standard pulse protocol used in daily clinical practice for electrochemotherapy can also be used for effective GET.

**Materials and methods:**

Experiments were performed *in vitro* in a tumor (B16F10) and two normal tissue cell lines (C2C12 myoblasts and L929 fibroblasts). Four different GET protocols, three using monopolar electric pulses and one bipolar electric pulses, were tested for the GET of plasmid DNA, which codes for green fluorescent protein *in vitro*. In addition, two GET protocols were chosen for *in vivo* tumor and muscle transfection.

**Results:**

Two GET protocols using monopolar electric pulses of different voltages delivered at 1 Hz transfected B16F10 tumor cells significantly better than normal cells. GET4 protocol, which uses monopolar electric pulses at 5 kHz, again transfected the B16F10 tumor cells significantly better, but the difference to the C2C12 myoblast cells was not significant. Compared with other GET protocols, GET3 using bipolar electric pulses at 1 Hz was significantly less effective. Both the GET2 (1 Hz) and GET4 (5 kHz) protocols resulted in similar tumor transfection efficiencies, whereas only the GET4 protocol was effective for muscle transfection *in vivo*.

**Conclusions:**

Our study demonstrated the efficient transfection of tumors and muscles with the GET4 pulse protocol, which is used clinically for electrochemotherapy. The use of this protocol could enable simultaneous electrochemotherapy and GET of the therapeutic gene in one session, which will significantly shorten the procedure and thus will be more tolerable for patients.

## Introduction

In recent years, various gene therapy strategies have been developed for cancer treatment. Although viral vectors are the most efficient methods for gene delivery, they have a limited loading capacity and could potentially be immunogenic and mutagenic.^1,2^ Therefore, nonviral delivery methods have been developed, especially for *in vivo* use. One of these methods is electroporation, which is called gene electrotransfer (GET), when it is used in the context of nucleic acid delivery.

Electroporation facilitates the introduction of various molecules into target cells or tissues. The exposure of cells or tissues to an external electric field results in the formation of transient hydrophilic structures in the cell membrane that enable the entry of molecules that are hydrophilic or too large to easily cross the plasma membrane.^3^ In cancer research and treatment, electroporation is widely used in electrochemotherapy to enhance the delivery of the cytotoxic molecules bleomycin and cisplatin.^3–7^ Today, electrochemotherapy is an established local ablative therapy for the treatment of cutaneous and deep-seated tumors of various histologies.^8–10^ Electroporation, i.e., GET, can also be used for the intracellular delivery of large molecules, such as plasmid DNA, siRNA, and mRNA, into target cells. Numerous preclinical and clinical studies have investigated electroporation-based gene delivery for cancer treatment. These include antiangiogenic therapy, cancer vaccines and immunotherapy.^11–14^

The ability of molecules to enter cells by electroporation depends largely on their size. Large molecules, such as plasmid DNA, therefore require more complex uptake mechanisms, including both endocytosis and direct transport through electropores.^15^ In addition, GET varies by cell type. For successful gene therapy, developing an efficient, safe, and tissue-specific gene delivery system is crucial. Therefore, optimizing the delivery protocol is very important. For such studies, fluorescent reporter genes, such as green fluorescent protein (GFP) or red fluorescent protein (DsRed), are commonly used to provide direct evidence of transgene uptake and expression.^16^

Several studies have investigated the parameters for both electrochemotherapy and GET.^17–19^ In recent years, several studies have combined electrochemotherapy and GET. In most cases, two different pulse protocols are used, each for a specific treatment.^20,21^ The aim of our current study was to test whether the standard electrochemotherapy pulse protocol, which is used in everyday clinical practice, could also be used for effective gene delivery, thus enabling the introduction of cytotoxic molecules and therapeutic genes with a single pulse protocol and electrodes in one session.

## Materials and methods

### Cell lines

The murine melanoma B16F10, myoblast C2C12 and fibroblast L929 cell lines (American Type Culture Collection, Manassas, VA, USA) were cultured in Dulbecco′s Modified Eagle Medium (DMEM; Gibco, Thermo Fisher Scientific, Waltham, USA), supplemented with 5% (v/v) fetal bovine serum (FBS; Gibco), 10 ml/L L-glutamine (GlutaMAX; Gibco) and 1% (v/v) penicillin–streptomycin (stock solution, 10,000 U/mL, Gibco). The cells were maintained in a 5% CO_2_ humidified incubator at 37 °C. The cells were routinely tested and confirmed to be free from mycoplasma infection, using the MycoAlertTM PLUS Mycoplasma Detection Kit (Lonza Group Ltd., Basel, Switzerland).

### Plasmid

The plasmid pEGFP-N1 (pGFP, Clontech, Takara Bio, Shiga, Japan), which encodes enhanced green fluorescent protein (EGFP), was amplified in competent *E. coli*, purified with the Endo Free Plasmid Mega Kit (Qiagen, Hilden, Germany) according to the manufacturer′s protocol and dissolved in endotoxin-free water at a concentration of 1 mg/mL. The concentration of the plasmid was measured with a Qubit 3.0 fluorometer (Thermo Fisher Scientific). The purity of the isolated plasmids was determined using the Take3™ Micro-Volume Plate (BioTek Instruments, Winooski, VT, USA) by measuring the 260/230 and 260/280 absorbance ratios. In addition, the identity of the individual plasmids was verified using restriction analysis.

### *In vitro* GET

The cells were collected and centrifuged and then resuspended in cold electroporation buffer (125 mM sucrose, 10 mM K_2_HPO_4_, 2.5 mM KH_2_PO_4_, 2 mM MgCl_2_ × 6 H_2_O) at a concentration of 25 × 10^6^ cells/mL. The cells were mixed with the plasmid mixture (1 mg/mL) at a ratio of 1:0.2, and 50 μL (1× 10^6^ cells) was electroporated by an electric pulse generator (GeneDrive, IGEA S.p.A., Carpi, Italy). The GeneDrive electroporator was a generous gift from IGEA S.p.A. The mixture was pipetted between two parallel stainless-steel plate electrodes with a distance of 2.5 mm. Four pulse protocols were used (Table 1). After electroporation, the cells were incubated for 5 min in 24-well low-attachment plates (Corning Incorporated, Corning, NY, USA), and then, 1 mL of complete DMEM was added without phenol red. The cells were seeded for particular assays as described below (cytotoxicity and transfection assays).

**TABLE 1.** Pulse parameters for different gene electrotransfer (GET) protocols for *in vitro* cell transfection

Experimental groups (protocol name)	Voltage (V)	Pulse duration (μs)	No. of pulses	Pulse direction	Frequency (Hz)
**GET1**	250	100	8	unipolar	1
**GET2**	300	100	8	unipolar	1
**GET3**	300	100	3	bipolar	1
**GET4**	300	100	8	unipolar	5000

### *In vitro* transfection assay

The cells were plated on a 96-well plate; 10000 cells/well from each experimental group and 5000 cells/well for control non-electroporated cells. The cells were imaged, and fluorescence was measured in real time with a Cytation 1 multimodal reader (BioTek Instruments) for 24 h every two hours. The cells were incubated at 37°C and 5% CO_2_ throughout the experiment. On the basis of the preliminary transfection results, the first image was captured 2 h after GET.

The analysis was performed using Gen5 data analysis software (BioTek Instruments). Images were acquired using a 4′ objective and imaging filter cubes of GFP (excitation at 469/35 nm and emission at 525/39 nm) for the detection of GFP. Two high-contrast brightfield images were also acquired: an in-focus image for reference and a defocused image for cell counting. Each well was focused with a laser autofocus cube. The exposure settings of the camera were optimized and kept the same for each experiment. The defocused image from the brightfield channel was processed with a black background and a 20 μm rolling ball. This increased the contrast and reduced each cell to a single bright spot. The GFP images were also processed with a black background and a 100 μm rolling ball to remove background fluorescence. The images were analyzed by masking the high-contrast defocused brightfield image and extending the brightfield mask to capture the GFP signal. The transfection efficiency was calculated by dividing the number of GFP-positive cells by the total number of cells in the brightfield image. The fluorescence intensity was determined by the mean of the intensity of all GFP-positive pixels in a field of view.

### Cytotoxicity assay

B16F10, C2C12 and L929 cells from each experimental group were plated in 100 μL of medium in 96-well plates (Corning Inc.) at a density of 1500 cells per well. The cells were then incubated at 37°C and 5% CO_2_ for 72 h. Ten microliters of Presto Blue® reagent (Thermo Fisher Scientific) was added to the wells, and the fluorescence intensity was measured one hour later with a Cytation 1 Multimodal Reader (BioTek Instruments). The results were plotted as the percentage of viable cells compared with that of the nonelectroporated control group.

### Animals and tumors

Ten-week-old female C57Bl/6NCrl (Charles River, Lecco, Italy) were used for the animal experiments. For tumor formation 100 mL of B16F10 cells in saline were injected into the flanks of the mice at a concentration of 3 × 10^5^ cells/mL. The mice were housed under specific pathogen-free conditions in a carousel mouse IVC rack system (Animal Care Systems Inc., Revere Parkway, USA) at a relative humidity of 55 ± 10%, a temperature of 20–24°C and a 12 h light/dark cycle. Food and water were provided *ad libitum*. The experimental procedures were performed in compliance with the guidelines for animal experiments of the EU directive (2010/63/EU) and with permission from the Veterinary Administration of the Ministry of Agriculture, Forestry and Food of the Republic of Slovenia (permission no. U34401-3/2022/17). The experimental procedures followed the PREPARE, OBSERVE and ARRIVE guidelines.^22–24^

### *In vivo* GET

Electroporation of the tumor and muscle tissue was performed with an electric pulse generator Cliniporator™ (IGEA s.r.l.).

#### Intratumoral GET

When the tumors reached 50 mm^3^, 30 μg of pGFP (1 mg/mL) was intratumorally injected, and after 5 minutes, GET was performed with two parallel stainless-steel electrodes with a 6 mm distance between them. Ultrasound conductive gel (Ultraschall Gel, P.J. Dahlhausen, & Co. GmbH, Koln, DE) was used to ensure good contact between the electrodes and the skin. GET was performed by two different protocols previously used in *in vitro* experiments: GET2 and GET4. Briefly, the GET consisted of eight 100 μs square-wave electric pulses, with an amplitude of 720 V (for 6 mm electrodes) and a frequency of 1 Hz (GET2) or 5 kHz (GET4), respectively.

#### Intramuscular GET

Thirty microliters of pGFP (1 mg/mL) were injected into the *tibialis cranialis* muscle, and then the leg was placed between two parallel stainless-steel electrodes with a 6 mm distance between them. Three different GET protocols were used: GET2 and GET4. As a control, a standard GET protocol for muscle transfection was also used, consisting of a combination of 1 high-voltage pulse, 360 V, pulse duration 100 μs, frequency 1 Hz) and 4 low-voltage pulses, 48 V, pulse duration 100 ms, frequency 1 Hz. There was a 1 s pause between high- and low-voltage pulses, and after the second low-voltage pulse, the electrodes were turned 180°.^18^

### *In vivo* transfection assay

After 48 h, the tumors and muscle were excised and fixed in 4% paraformaldehyde (PFA; Alfa Aesar) overnight. The samples were then incubated in 30% sucrose for 24 h, embedded in optimal cutting temperature compound (OCT compound, VWR International, Radnor, PA, US) and snap frozen in liquid nitrogen. Fourteen-micron-thick tumor sections were cut using a Leica CM1850 cryostat, dried for 10 min at 37 °C and washed for 5 min in 1′ PBS. All the sections were stained for 10 min with Hoechst solution (3 μg/mL) and then washed for 5 min in 1′ PBS. The slides were then mounted with ProLong™ Glass Antifade Mountant (Thermo Fisher Scientific) and covered with cover glass, and the edges were sealed with nail polish. The slides were then imaged using a Zeiss Axio Observer fluorescence microscope (Carl Zeiss, Oberkochen, Germay) equipped with Colibri 7 LED Light source (UV (385nm), Violet (430nm), Blue (475nm), Green (555nm), Yellow (590nm), Red (630nm), Far Red (735nm)) and a Hamamatsu Orca Flash 4.0 V3 camera. Emitted light was imaged through the following filters: Filter Set HE BFP shift free (Carl Zeiss) for Hoechst 33342 (nuclei), filter set 38 HE eGFP shift free (Carl Zeiss) for EGFP. Images were then visualized in Imaris software (Oxford Instruments, Abingdon, UK).

### Statistical analysis

The comparison of means of more than two groups was statistically evaluated by one-way analysis of variance (one-way ANOVA) followed by Dunnett′s or Tukey′s multiple comparisons test. A p value of < 0.05 was considered to indicate statistical significance. The values on the graphs are presented as the mean (AM) ± standard error of the mean (SE). All *in vitro* experiments were performed three times with six technical replicates. *In vivo* experiments were performed in six muscles and six tumors for each experimental protocol. For statistical analysis and preparation of graphs, GraphPad Prism 10.4.1 (La Jolla, CA, USA) was used.

## Results

### The transfection efficiency of a cell line depends on the pulse parameter protocol

**FIGURE 1.**
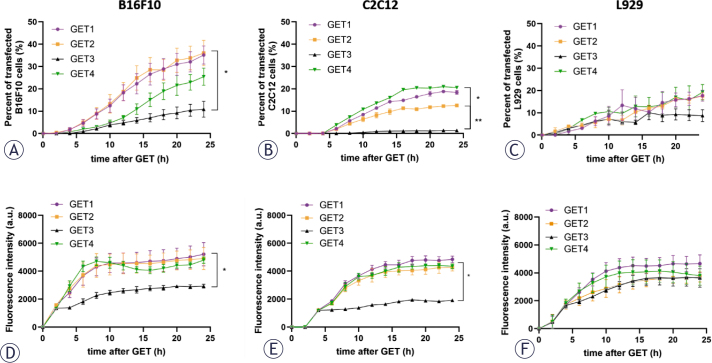
The transfection efficiency timeline of different cell lines according to the gene electrotransfer (GET) protocol. Percent of transfected B16F10 **(A)**, C2C12 **(B)** and L929 **(C)** cells and fluorescence intensity of B16F10 **(D)**, C2C12 **(E)** and L929 **(F)** cells. *p < 0.05, statistically significant difference in GET1/GET2 compared with GET4 (A); *p < 0.05, statistically significant difference in GET1/GET4 compared with GET3 and GET2 (B); **p < 0.05, statistically significant difference in GET2 compared with GET3 (B); *p < 0.05, statistically significant difference in GET1/GET2/GET4 compared with GET4 (D and E)

The transfection efficiency of three different cell lines, i.e., B16F10 melanoma, C2C12 myoblasts and L929 fibroblasts, was measured as the percentage of transfected cells as well as the fluorescence intensity. To better understand the dynamics of transfection, the transfection efficiency timeline was measured (Figure 1). After two hours, the transfection was detected by an increase in the fluorescence intensity signal. Thereafter, it steadily increased and reached a plateau approximately 6–10 hours later.

In two tested cell lines, B16F10 and C2C12, we confirmed previous observations that the transfection efficiency of a cell line depends on the GET protocol. In both cell lines, the use of GET1, GET2 and GET4 resulted in higher percentage of transfected cells as well as fluorescence intensity than did the use of GET3 (Figure 1A, D, B, E). The transfection efficiency differed between the cell lines. The highest level of transfection was observed in B16F10 melanoma cells, followed by C2C12 and L929 cells (Figure 1A, B, C). The fluorescence intensity patterns were similar for both B16F10 and C2C12; compared with those of GET3, the fluorescence intensities of GET1, GET2 and GET4 were significantly higher (Figure 1D, E). Thus, GET3 with bipolar electric pulses was significantly less effective than monopolar GET1, GET2 and GET4 pulses in B16F10 and C2C12 cells. In the L929 fibroblast line, the transfection efficiency did not depend on the GET protocol, either in terms of the percentage of transfected cells or the fluorescence intensity.

**FIGURE 2.**
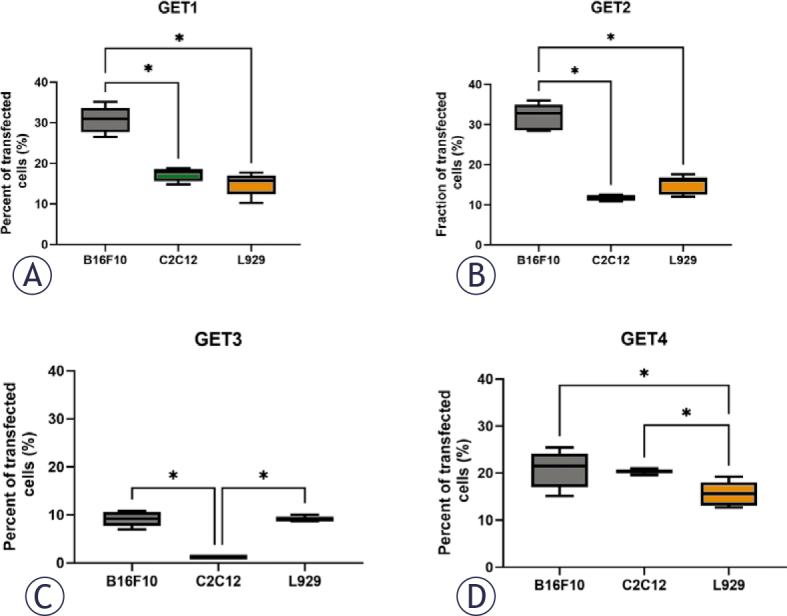
Transfection efficiency of different pulse parameter protocols in three different cell lines. Percent of transfected tumor (B16F10) and normal (C2C12, L929) cells after GET1 **(A)**, GET2 **(B)**, GET3 **(C)** and GET4 **(D)** treatment. *p < 0.05 indicates a statistically significant difference; GET = gene electrotransfer

The percentage of transfected cells at 24 h was used to compare the transfection efficiency among the three cell lines using four different GET protocols (Figure 2). This approach was used to select the most efficient GET protocol for specific cell types. The pulse parameters of GET1 and GET2 transfected B16F10 tumor cells significantly better than normal cells, whereas after GET4, B16F10 tumor cells were again significantly better transfected than L929 cells, but the difference in C2C12 cells was not significant. Compared with C2C12 myoblast cells, B16F10 melanoma and L929 fibroblasts were significantly more effectively transfected with GET3 (Figure 2).

**FIGURE 3.**
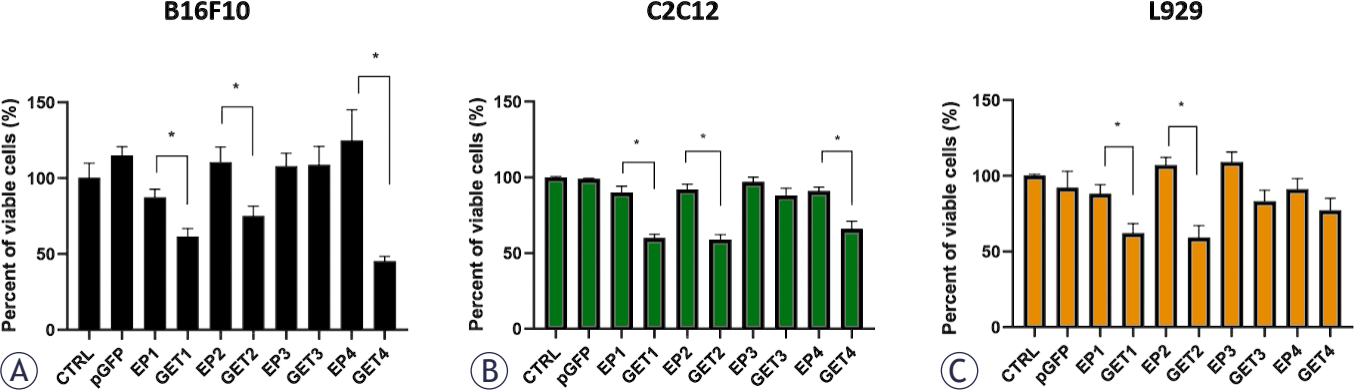
Cell survival after different pulse parameters and gene electrotransfer (GET) in B16F10 **(A)**, C2C12 **(B)** and L929 **(C)** cells. * = p < 0.05 indicates a statistically significant difference; EP = electroporation only; pGFP = plasmid only, which encodes the green fluorescent protein

The cytotoxicity of electric pulses (EPs) alone and the GET of cells in the presence of the plasmid were tested in all three cell lines (Figure 3). The pattern of cell survival, as measured by the percentage of viable cells, was similar among the three cell lines. The application of EPs alone did not cause any significant reduction in cell viability, and up to 15% of the cells died.

In contrast, GET1, GET2 and GET4 significantly reduced cell survival compared with the corresponding EP protocol in B16F10 and C2C12 cells. In L929 cells, only GET1 and GET2 significantly reduced cell survival, whereas GET4 did not. GET3 did not cause a cytotoxic effect in any of the cell lines (Figure 3 A, B, C).

### Tumor and muscle transfection

For *in vivo* transfection efficiency, two GET protocols, GET2 and GET4, were selected on the basis of the *in vitro* results. They differ only in repetition frequency: GET2 has 1 Hz, and GET4 has 5 kHz. GET2 had the greatest effect on the transfection efficiency (approximately 35%) of B16F10 melanoma cells but had a lower effect (approximately 15%) on C2C12 myoblasts and L929 fibroblasts. The GET4 pulse protocol had an approximately 20% transfection efficiency in tumor cells but a similar transfection efficiency in muscle cells. For fibroblasts, however, the efficiency was significantly lower but was still ~ 15% (Figure 2).

**FIGURE 4.**
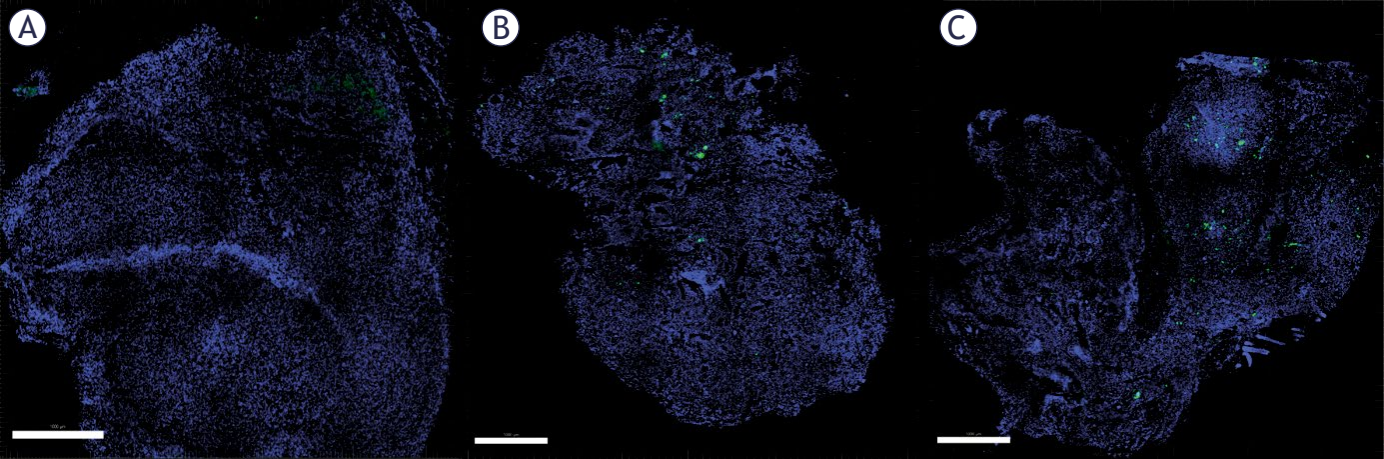
Transfection of B16F10 tumors. Untreated control tumors **(A)**, GET2 **(B)** and GET4 **(C)** tumors. Nuclei are stained blue with Hoechst. The transfected cells are presented in green, indicating transfection with the GFP plasmid. Scale bar = 1000 μm

Frozen tumor and muscle sections were first stained with Hoechst for nuclei staining to visualize the tissue better. GFP was more highly localized in the rims of the B16F10 tumors (Figure 4). Both the GET2 (1 Hz) and GET4 (5 kHz) protocols resulted in similar transfection efficiencies; only a small percentage of the tumor cells were transfected. Compared with these data, similar transfection rates (approximately 2%) were reported in our previous studies in which millisecond pulses (600 V/cm, 5 ms, 1 Hz) were used.^25^

**FIGURE 5.**
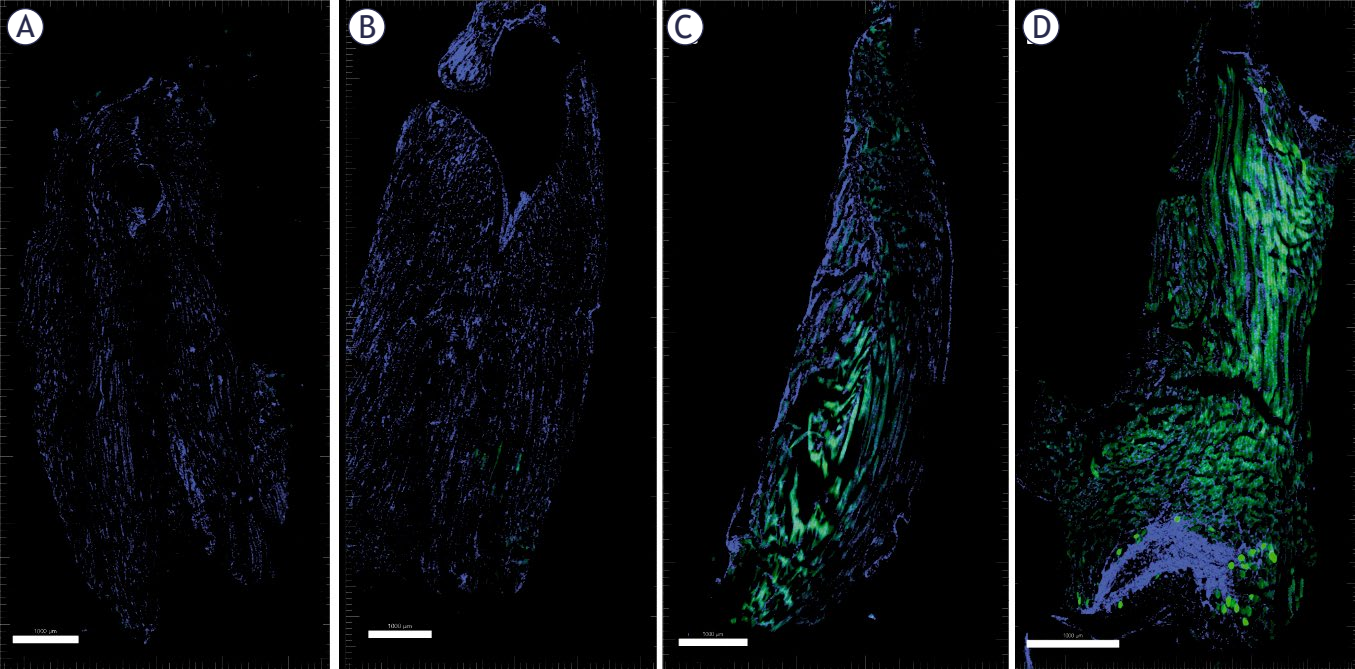
Transfection of muscle. Control **(A)**, GET2 **(B)**, GET4 **(C)** and 1HV-4LV **(D)**. The nuclei are stained blue with Hoechst. The transfected cells are presented in green, indicating transfection with the GFP plasmid. Scale bar = 1000 μm.

In contrast to tumors, efficient transfection was obtained in muscle only after the GET4 protocol. However, the transfection efficiency was not as effective as that of standard GET with the 1HV-4LV pulse protocol, which has been optimized for GET into muscle in previous studies and served as a positive control in the current study (Figure 5D).

## Discussion

In the present study, we demonstrated that the GET of plasmid DNA could also result in high transfection efficiency with a standard electrochemotherapy pulse parameter protocol.^26^

First, we tested different pulse parameter protocols in one tumor (B16F10 melanoma) and two normal cell lines *in vitro* (C2C12 myoblasts and L929 fibroblasts). As mentioned, GET protocols were selected on the basis of clinically used pulses. GET4 has identical pulse parameters, which are used in electrochemotherapy: 8 pulses, 960 V (for the 8 mm electrode), and a 100 μs pulse with a frequency of 5 kHz.^3,26^ The voltage was calculated for the 2.4 mm electrodes used for the *in vitro* experiments and reached 300 V. In the first ESOPE clinical trial, the effectiveness of such 5 kHz electrochemotherapy pulses was compared with that of 1 Hz pulses; therefore, we also tested pulses with a frequency of 1 Hz, GET2.^27^ These parameters are used in clinical settings for electrochemotherapy with plate electrodes. The Cliniporator has another option, for electrochemotherapy, with needle electrodes, where the voltage-to-distance ratio is set to 1000 V/cm and the GET1 parameter mimics this setting but with a frequency of 1 Hz.^3^ Bipolar pulses, GET3s, are not yet used in clinical settings but have been proposed as alternative protocols since they are less painful and could cause less muscle contraction.^28–30^

As expected, the transfection efficiency of a cell line normally depends on the pulse parameter protocol.^16,31,32^ However, in our study, this was confirmed for B16F10 and C2C12 cells but not for L929 cells, as there was no significant difference in the transfection of L929 cells after GET with different pulse protocols. As previously reported in several studies, GET was significantly cytotoxic to all cell lines. The exposure of cells to EP alone was not significantly toxic to either cell line, indicating that the major reason for the cytotoxic effect could be the introduction of exogenous DNA into the cytosol, which triggers the cytosolic DNA sensor pathway.^33,34^ The transfection efficiency of B16F10 cells was similar (approximately 35%) to that reported in previous studies with millisecond pulses, indicating that shorter pulses with higher voltage-to-distance ratios can efficiently transfect cells.^31,35^ *In vitro,%* we previously reported that higher percent of C2C12 cells were transfected when 5 kHz (GET4) pulses were used than when 1 Hz pulses (GET2) were used. The use of bipolar pulses *in vitro* resulted in lower transfection efficiency, and further *in vivo* studies are needed to explore the transfection potential of such pulses better.

Our research focused on standard electrochemotherapy pulses for *in vivo* transfection. In recent years, preclinical studies have suggested that combining electrochemotherapy and gene therapy could lead to a better local response and could also result in an abscopal effect.^20^ Electrochemotherapy of tumors could serve as an *in-situ* vaccination, which can be boosted by electrotransfer of transgene that encodes immunostimulat.^36^

Clinical electrochemotherapy pulses have already been investigated for the introduction of plasmid DNA encoding human (phIL12) and mouse IL-12 (pmIL12) into cells *in vitro*. The suitability of the plasmids was evaluated by assessing the expression levels of IL-12 mRNA and protein, the biological activity of IL-12, and the plasmid copy number *in vitro* in human (FaDu) and murine (CT26) carcinoma cell lines.^37^ Later, the murine analog pmIL12 was tested in an *in vivo* tumor model*,%* where the antitumor effectiveness of the protein accompanied by immune cell infiltration was demonstrated. The plasmid was distributed throughout the body, but the amount decreased over time. The treatment did not cause any systemic toxicity. Overall, the results of the nonclinical evaluation demonstrated the safety and efficacy of the pmIL12/phIL12 GET.^38^ These nonclinical *in vitro* and *in vivo* results formed the foundation for the first-in-human clinical study. The study was a dose-escalating phase I study that demonstrated the safety and feasibility of the treatment. Histological analysis revealed the expression of IL-12, immune cell infiltration and downstream IFN-γ production in tumor samples.^39^ Another study of IL-12 plasmid DNA transfection into porcine skin provided supportive data for efficient gene delivery with electrochemotherapy pulses.^40^ Here, we demonstrated that B16F10 melanoma tumors could also be efficiently transfected with standard electrochemotherapy pulses, thus providing a basis for expanding the use of IL-12 immune therapy to other tumor types, such as melanoma, which is a tumor type often treated with electrochemotherapy, especially in-transit metastases.^41^

To the best of our knowledge, GET of muscle with electrochemotherapy pulses has not yet been performed. One research group used six pulses with a voltage-to-distance ratio of 1300 V/cm, a pulse duration of 100 μs and a frequency of 4 Hz, but the experiments on muscle cells were performed only *in vitro*.^42^ A phase I clinical study of plasmid AMEP GET into muscle has been performed with Cliniporator, but the pulse protocol consists of one high-voltage pulse and one low-voltage pulse.^43^ This protocol was used in our current study as a control protocol, and according to our present results, this GET protocol yielded high transfection efficiency, which was higher than that of the GET4 protocol; therefore, this protocol is not suitable for use in vaccination studies. In the vaccination study published by Aurisicchio *et al*., intramuscular injection followed by electroporation with a low voltage of 40 V and 4 pulses of 5 ms, separated by 5 ms intervals, was performed via four single-use, sterile intramuscular needle electrodes included in a newly designed electroporation system gun connected to the Cliniporator.^44^

The combined treatment of electrochemotherapy and IL-12 GET has already been used in clinical studies to treat various spontaneous tumors in client-owned dogs. A recent clinical trial in spontaneous canine mast cell tumors compared electrochemotherapy alone or in combination with either intratumoral (i.t.) or peritumoral (peri.t.), reported significantly better local tumor control in the electrochemotherapy + GET i.t. group than in the electrochemotherapy + GET i.t. group. or electrochemotherapy groups. In addition, the disease-free interval and progression-free survival were significantly longer in the electrochemotherapy + GET i.t. group than in the other two groups.^45^

Therefore, the results of our study, together with the abovementioned results of studies in veterinary oncology, support concomitant GET and electrochemotherapy treatment in one session with the same pulse protocol and electrodes, as it results in efficient transfection of tumors. Since electrochemotherapy has different response rates in different tumor types^46^ the addition of concomitant immunomodulatory gene therapy could increase the percentage of complete responses or flip the local effect into a locoregional or systemic abscopal effect.
